# A Rare Case of Orbital Adenoid Cystic Carcinoma Mimicking as Optic Nerve Glioma

**DOI:** 10.7759/cureus.25863

**Published:** 2022-06-12

**Authors:** Abhishek GU, Sachin Daigavane

**Affiliations:** 1 Department of Ophthalmology, Jawaharlal Nehru Medical College, Datta Meghe Institute of Medical Sciences University, Sawangi, IND

**Keywords:** relative afferent pupillary defect, orbito-zygomatic craniotomy, sudden diminution of vision, optic nerve glioma, adenoid cystic carcinoma

## Abstract

Adenoid cystic carcinoma is an uncommon epithelial cell tumour that usually originates from glands. They arise from the upper respiratory tract, lungs, mammary glands, and skin, but most commonly from the salivary glands and lacrimal glands. Our article reports a 53-year-old individual presenting with a history of diminution of visual acuity over the past one and a half months, also associated with a right-sided headache and throbbing eye pain not relieved by medication. Examination revealed right axial proptosis, ptosis, and visual acuity of 6/36, right afferent pupillary defect (RAPD), restriction of ocular movements in supraduction, dextroelevation, and abduction. The fellow eye was completely normal. MRI revealed an enhancing lesion in the retrobulbar area of the right orbit indicative of optic nerve glioma of stage 2. The patient underwent orbito-zygomatic craniotomy with subtotal excision of the mass by a neurosurgeon. Following surgery, histopathological examination of the excised tumour revealed features consistent with adenoid cystic carcinoma. On the third post-operative day, the subject's vision improved to counting fingers at 3 metres, and extraocular movements were regained.

## Introduction

Adenoid cystic carcinoma (ACC) is an epithelial cell tumour that is locally invasive, occurring in the neck region and originating from the salivary glands and lacrimal glands [1]. Those arising from the lacrimal gland are usually present as a super temporal orbital mass, and those from the salivary gland are usually present in the nasal cavity and paranasal sinuses [1-4]. Common sites of origin for ACC are the nasopharynx, salivary, and lacrimal glands, but for intracranial ACC it is the skull base [3]. In spite of having a low probability of distant metastasis, the most common site of metastasis is the lungs [2]. Having been diagnosed late, adenoid cystic carcinoma lies in close proximity to vital structures like the brain, dura, and cranial nerves; hence, the chances of adequate surgical resection of such tumours are quite low. ACC of paranasal sinuses can spread by perineural route [2]. Intracranial ACC can mimic meningiomas [5]. There is evidence of ACC involving the cavernous sinus as well [6]. This feature of ACC demands neurosurgery intervention and prompt surgical management.

Incidences of orbital adenoid cystic carcinoma are lower [7-9]. The orbital ACC arises from the lacrimal gland. Primary ACC of the orbit of extra lacrimal origin is quite rare [8,9]. Due to its peculiar location, patients will manifest ophthalmological complaints due to cranial nerve involvement. Such cases with primary ophthalmic manifestations may be diagnosed as any other retrobulbar tumour on initial assessment because they tend to have similar presentations. We report one such rare incidence of the orbital ACC that mimicked an optic nerve glioma.

## Case presentation

A 53-year-old individual presented to the eye OPD with complaints of gradual diminution of vision in the right eye for one and a half months. The diminution of vision had a subacute onset, was rapidly progressive, and progressed to complete loss of vision within one and a half months. It was also associated with pain in the eye, which was throbbing in character, not relieved by medication, radiating to the forehead and jaw. The patient also complained of a right-sided continuous severe headache. There was no history of proptosis/redness/watering of eyes/photophobia, no history of any ocular surgery in the past, history of trauma to the eyes, and no history of radiation exposure. The patient’s medical history was not significant for hypertension, hyperthyroidism, or diabetes mellitus.

Examination

**Figure 1:**
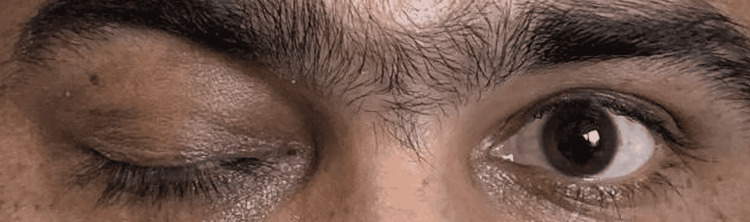
Right ptosis Right eye shows complete ptosis

On examination, there was uniocular (right) axial proptosis, ptosis (Figure 1 showing ptosis), and visual acuity was 6/36 with a projection of rays being accurate in all quadrants.

**Figure 2:**
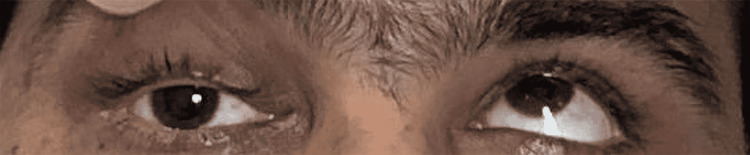
Right eye in elevation Image shows restriction of elevation in right eye while left eye elevates normally

**Figure 3:**
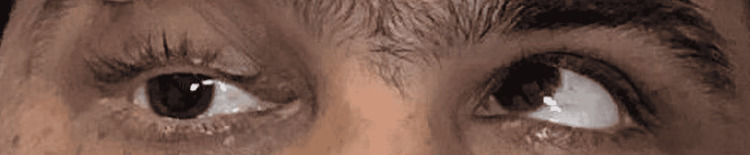
Right eye in dextroelevation Image shows restriction of right eye in dextroelevation

**Figure 4:**
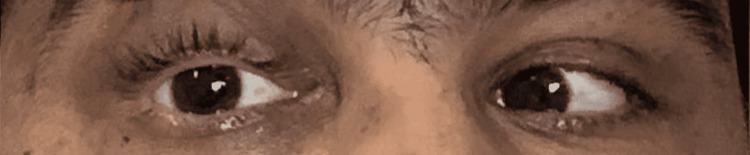
Right eye in abduction Image shows restriction of abduction movement in right eye

There was a restriction of ocular movements of the right eye in supraduction (Figure 2), dextroelevation (Figure 3), and abduction (Figure 4).

The swinging flashlight test revealed a right afferent pupillary defect (RAPD). The left eye did not show any RAPD, ocular movements in the right eye were free and full in all directions of gaze, and visual acuity was 6/6.

Radiological investigations

**Figure 5:**
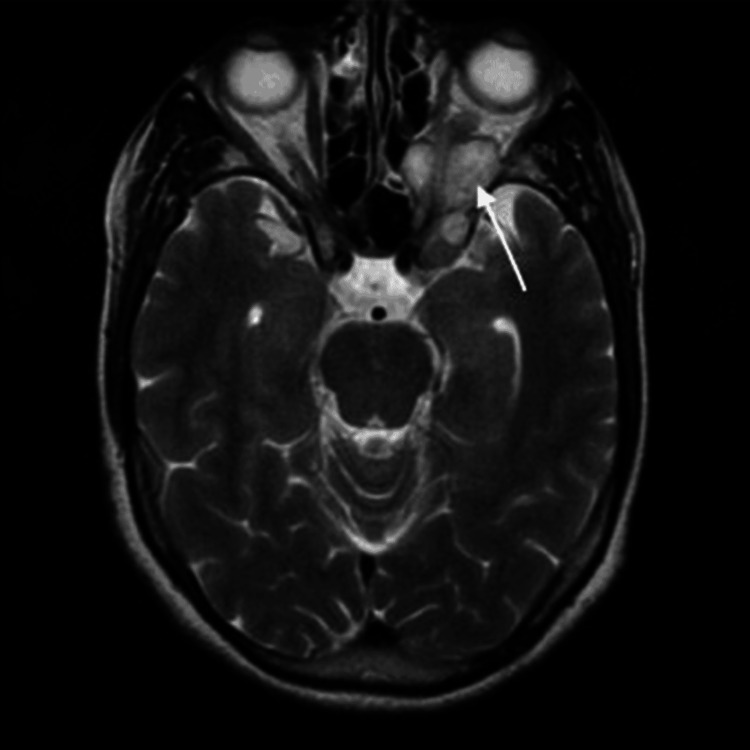
MRI scan of brain and orbit showing smooth enhancing lesion in retrobulbar region Arrow shows hyperintense lesion along the optic nerve (arrow), extending to middle cranial fossa

**Figure 6:**
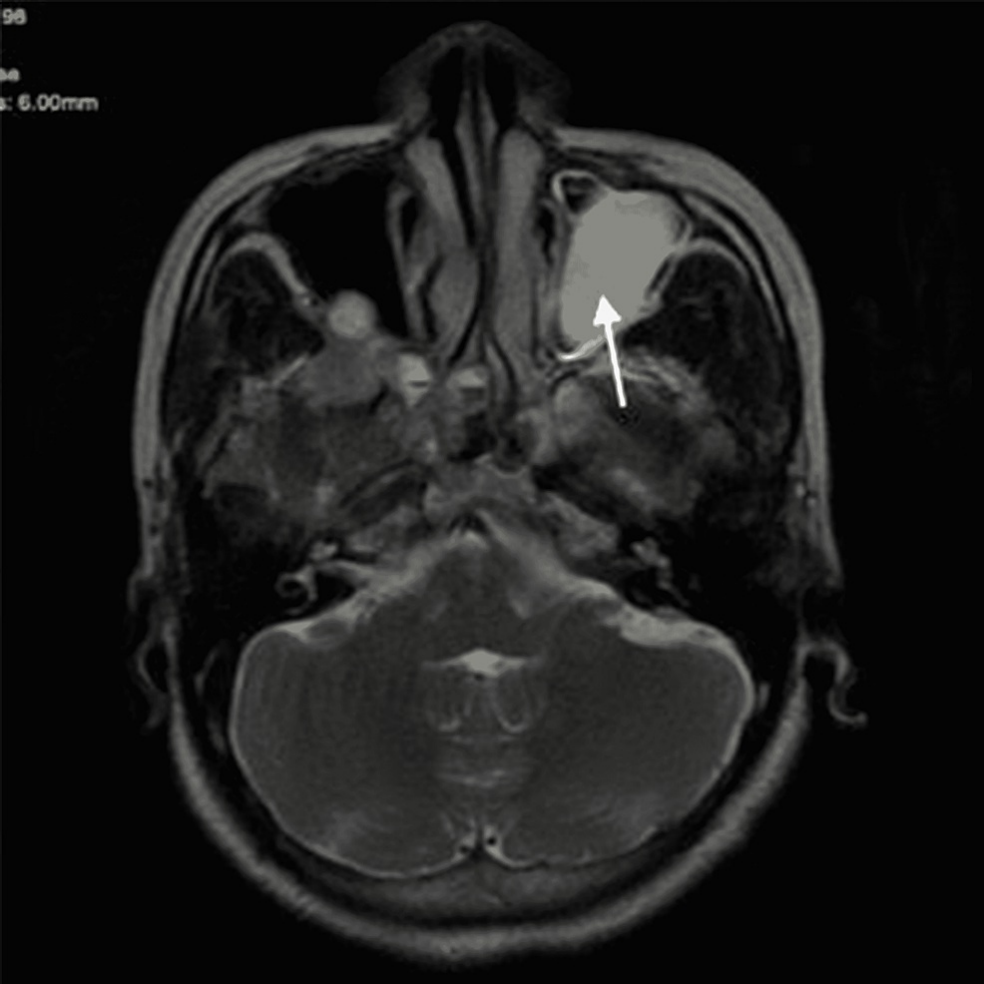
MRI scan of brain and orbit Arrow indicating enhancing lesion in the retrobulbar aspect of orbital apex

MRI of the brain with orbits demonstrated a smooth enhancing lesion in the retrobulbar region of the right orbit at the apex (Figures 5-6), which was hyperintense on T2 images, causing widening of the optic foramen, medially compressing posterior ethmoid air cells, and extending in the middle cranial fossa (Figure 5) along the optic chiasma on the right with the loss of fat plane between the cavernous sinus, inferior rectus muscle, and optic nerve (Figure 6), suggestive of optic nerve glioma stage 2.

Discussion of management

As the patient was a suspected case of optic nerve glioma, she was operated on by a neurosurgeon for orbito-zygomatic craniotomy with subtotal excision of the mass. Intra-operatively, the tumour was soft to firm in consistency with an adherent capsule. During excision, it was found that the mass was separate from the optic nerve.

Histopathology

**Figure 7:**
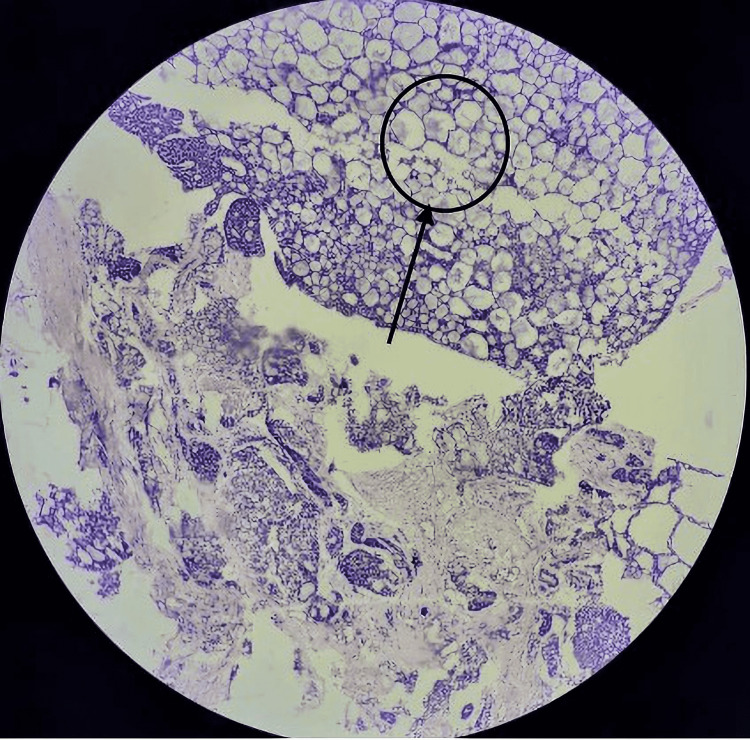
Histopathological examination of specimen (low power, 10×) Arrow shows tumour cells arranged in tubular pattern indicative of adenoid cystic carcinoma

Histopathological examination of the excised tumour revealed a section stained in H&E that shows tumor cells arranged in a tubular pattern (10×) (Figure 7).

**Figure 8:**
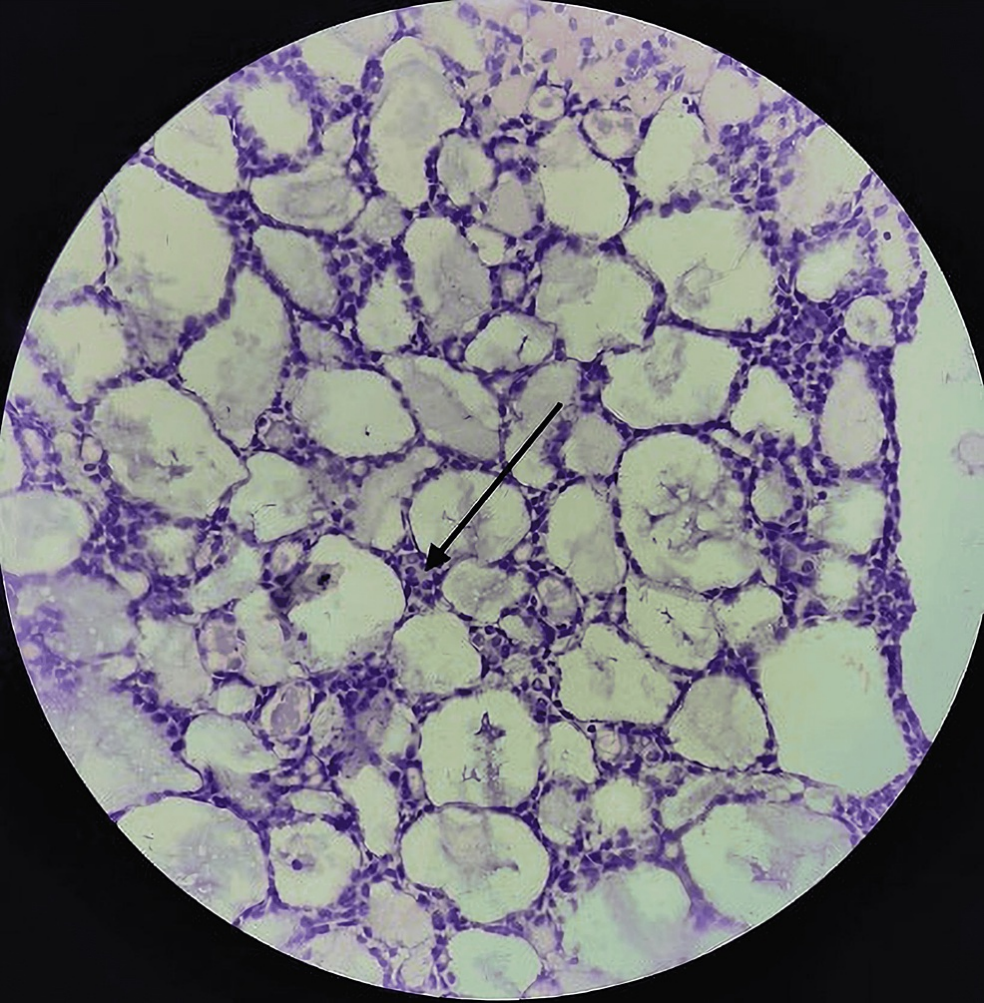
Histopathological slide of specimen (high power, 40×) arrow head points to basaloid tumour cells with hyperchromatic nuclei Arrow head points to basaloid tumour cells with hyperchromatic nuclei indicating adenoid cystic carcinoma

The section showed tumour cells arranged in a cribriform pattern filled with hyaline material. Individual tumor cells were basaloid and hyperchromatic with scanty cytoplasm diagnostic of adenoid cystic carcinoma (Figure 8).

As a diagnosis of adenoid cystic carcinoma was made, the patient was subjected to an MRI of the chest and abdomen to rule out secondary lesions, along with a cervical lymph node biopsy. However, all the tests came negative, indicating no evidence of secondaries. After three days following surgery, the subject's vision improved to counting fingers at 3 metres, and extraocular movements were regained. The relative afferent pupillary defect was no longer seen in the right eye. The patient was relieved of ocular pain and headache. Further long-term follow-up is required to make sure there is no recurrence or metastasis of the tumour.

## Discussion

The case highlights an unusual presentation of adenoid cystic carcinoma in retro-orbital region, mimicking optic nerve glioma on neuroimaging.

Adenoid cystic carcinoma, being an uncommon epithelial cell tumour usually originates from glands, most commonly salivary in the head and neck, but can also occur in other areas of the body [1-2]. Most ACCs arise from the salivary glands and the lacrimal gland, but they can also arise from the upper respiratory tract, lungs, mammary glands, and skin [3-6]. The most common presentation of orbital ACC is a superotemporal mass [7,8]. ACC with lacrimal tissue has a tendency to infiltrate its surroundings by retrograde perineural spread as well as lymphatic and hematogenous spread. Though orbital ACC has a common association with lacrimal tissue, further imaging modalities including CT, MRI and PET failed to recognise any possibility of a primary site. MRI studies in our case indicated optic nerve glioma stage 2, which also correlated clinically with a reduction of visual acuity. Later confirmation by histopathological examination confirmed that it was adenoid cystic carcinoma compressing the optic nerve and causing diminution of vision and the tumour of the optic nerve per se. Shields et al. [7] reported a case where ACC originated from ectopic lacrimal gland tissue in the medial orbital. Lin et al. [9] reported a case of orbital ACC with involvement of the inferior rectus but without any evidence of lacrimal tissue.

Very few cases of ACC have been recorded wherein there was no lacrimal gland involvement. Venkitaraman et al. [8] recorded ACC at the orbital apex in a 51-year-old male without any evidence of lacrimal tissue involvement. Adenoid cystic carcinoma can also arise outside the orbit. Arsene et al. [10] reported a case of bilateral ACC on the skull base extending into the cavernous sinus. The recurrence rate in the case of adenoid cystic carcinoma of the head and neck region is around 53% [11]. Hence, there is a need for follow-up.

Currently, the literature does not provide the most optimal treatment protocol for the management of adenoid cystic carcinoma. Treatment includes surgical resection and post-surgical radiotherapy. Newer therapies like plaque radiotherapy and intra-arterial cytoreductive chemotherapy have been employed to reduce disease recurrence and improve patient survival. However, we did not provide any such post-surgical radiotherapy or chemotherapy as our patient did not have any evidence of secondaries. Zhang et al. [12] reported two cases of atypical orbital adenoid cystic carcinoma without the involvement of the lacrimal gland. ACC presented as a retro-orbital mass extending into the orbital apex. One case was treated with orbital surgery and complete resection of the tumour. Immunohistochemistry showed invasion of optic nerve fibres by tumour cells. However, the second case showed large orbital adenoid cystic carcinoma involving all the extraocular muscles with optic nerve and orbital wall involvement. After knowing the unresectable nature of the tumour the patient aboned the treatment.

Shields et al. [13] also reported a case of orbital ACC mimicking a dermoid cyst radiologically in a 9-year-old boy. Initially, the mass was excised by the subconjunctival route and excision biopsy from orbital exploration revealed a small margin of positive for adenoid cystic carcinoma and was treated with plaque brachytherapy using radioactive iodine plaque. Macri et al. [14] reported a case of a 55-year-old male with progressively worsening diplopia, proptosis, ptosis, and restricted extraocular movements. MRI revealed an orbital lesion infiltrating both extraconally and intraconally extending into the inferior temporal fossa, pterygopalatine fossa, and pterygoid muscle. An excision biopsy revealed adenoid cystic carcinoma. They were not able to isolate the site of origin and did not mention any further lines of management in their case. Xia et al. [15] reported a case of a 66-year-old man presenting with left ocular swelling and left proptosis with restricted ocular movements. Incision biopsy was taken and immunohistochemistry revealed poorly differentiated adenocarcinoma of the orbit. The patient was treated with chemotherapy followed by radiotherapy, resulting in complete regression of the tumour. No surgical methods were used, unlike in our case.

## Conclusions

The case initially presented with symptoms of unilateral proptosis with severe loss of visual acuity. Both clinical examination and radiological diagnosis pointed to optic nerve glioma as the diagnosis. However, things took a turn when, during surgical excision, the lesion was seen separate from the optic nerve and compressing the optic nerve. Later histopathological diagnosis also confirmed it to be an adenoid cystic carcinoma.

The large tumour of ACC was compressing the optic nerve, and this was responsible for the loss of vision. The clinician should always take note of the varied clinical presentation of a rare tumour. This also shows the importance of histopathological examination for definitive diagnosis. One has to always keep in mind the possibility of orbital adenoid cystic carcinoma without any primary lesion, which is a rare presentation of the condition.
